# The Effect of Transborder Mobility on COVID-19 Incidences in Belgium

**DOI:** 10.3390/ijerph19169968

**Published:** 2022-08-12

**Authors:** Febe Brackx, Fien Vanongeval, Yessika Adelwin Natalia, Geert Molenberghs, Thérèse Steenberghen

**Affiliations:** 1 Spatial Applications Division, KU Leuven, 3000 Leuven, Belgium; 2 I-Biostat, Data Science Institute, Universiteit Hasselt, 3500 Hasselt, Belgium

**Keywords:** COVID-19, transborder mobility, case incidence

## Abstract

Belgium is a geographically small country bordered by The Netherlands, France, Germany, and Luxembourg, with intense transborder mobility, defined as mobility in the border regions with neighboring countries. It is therefore of interest to examine how the 14-day COVID-19 confirmed case incidence in the border regions is influenced by that of the adjacent regions in the neighboring countries and thus, whether and how it differs from that in the adjacent non-border regions within Belgium. To this end, the 14-day COVID-19 confirmed case incidence is studied at the level of Belgian provinces, well-defined border areas within Belgium, and adjacent regions in the neighboring countries. Auxiliary information encompasses work-related border traffic, travel rates, the proportion of people with a different nationality, the stringency index of the non-pharmaceutical interventions, and the degree of urbanization at the level of the municipality. Especially in transnational urbanized areas such as between the Belgian and Dutch provinces of Limburg and between the Belgian province of Antwerp and the Dutch province of North Brabant, the impact on incidence is visible, at least at some points in time, especially when the national incidences differ between neighboring countries. In contrast, the intra-Belgian language border regions show very little transborder impact on the incidence curves, except around the Brussels capital region, leading to various periods where the incidences are very different in the Dutch-speaking north and the French-speaking south of Belgium. Our findings suggest that while travel restrictions may be needed at some points during a pandemic, a more fine-grained approach than merely closing national borders may be considered. At the same time, in border regions with considerable transborder mobility, it is recommended to coordinate the non-pharmaceutical interventions between the authorities of the various countries overlapping with the border region. While this seems logical, there are clear counterexamples, e.g., where non-essential shops, restaurants, and bars are closed in one country but not in the neighboring country.

## 1. Introduction

During the COVID-19 pandemic that started in 2020 and is ongoing at the time of writing, non-pharmaceutical interventions (NPIs), including mask mandates, partial or full school and business closures, restrictions on private and public gatherings, domestic mobility restrictions, restrictions on public transport, stay-at-home orders, and international travel restrictions (entry restrictions, quarantining, isolation, and testing) were widely implemented in most countries to mitigate the spread of the disease [1]. In 2021, the KU Leuven Metaforum, an interdisciplinary think tank for societal debate, launched a short-term research project entitled “Construction of a Dashboard for the Evaluation of COVID-19 Policies”, to evaluate how NPIs deployed during the pandemic affect different components of wellbeing [2] for different target groups. The results of a literature review have been presented in a matrix, showing how the epidemiological effects of measures can be linked to trade-offs or synergies in other domains [3]. Assessment of the impact of NPIs should consider the various dimensions together. In this article, we investigate the impact of international travel restrictions in border regions where cross-border travel is intertwined with daily mobility.

International travel restrictions, as defined by the WHO [1], include entry restrictions, quarantining, and testing. Travel measures played an important role in the early transmission dynamics of the COVID-19 pandemic [4]. During the pandemic, travel bans lowered the number of COVID-19 imports overall, yet the effectiveness on individual countries varies and also depends on changes in the behavior of returning residents and citizens [5]. Tourism and travel, a major contributor to the service industry worldwide, was hard hit and was among the most severely impacted global industries [6]. The lifting of border control policies is key to global economic recovery [7]. Vaccination is considered a primary measure to contain the spread of COVID-19, and the increasing share of the world population vaccinated [8] is a strong argument for lifting international travel restrictions. However, at the same time, vaccination is less effective when travel restrictions are removed [9]. Assessing the impact of the international travel restrictions should therefore consider the specific local socio-economic and cultural conditions, as well as international travel in general and transborder travel in particular.

An analysis of nine EU countries, including Belgium, based on their population density and the degree of impact of the epidemic in the first six months of the pandemic, showed that the massive flow of personnel exchanges in the border area between The Netherlands, Belgium, France, and Germany (see Table 1 and Table 2) was one of the main reasons they ranked among the hardest-hit areas in Europe, in addition to the small size and high population density of The Netherlands and Belgium (NL: 41,543 km^2^ and 508 inhabitants/km^2^, BE: 30,688 km^2^ and 375 inhabitants/km^2^, EU: 109 inhabitants/km^2^). The study concluded that large land borders and well-developed transportation infrastructure between countries exacerbated the spread of COVID-19 [10].

In Belgium, internal borders also played a role in incidence. A nationwide survey conducted in September 2020 investigated the knowledge, understanding, and behavior of the adult population regarding COVID-19. Understanding (the logic of) the measures is essential to motivate people to adopt appropriate protective behavior. This understanding was lower in the French-speaking south of the country than in the Dutch-speaking north [11]. Transborder mobility effects depend not only on the characteristics of the neighboring country but also those of the Belgian border region. Limited intranational transborder mobility among regions may create border effects in incidences in a similar way to international travel restrictions.

The goal of this paper is to examine the incidence curves in Belgium’s border areas and contrast these with the corresponding curves in the neighboring areas across the border, as well as in Belgium. It is hypothesized that the relationship between the incidence curves in the border areas and the incidence curves across the border depend on the social and economic ties, the urbanization level of the area, and the health measures that were in place in both Belgium and the neighboring countries throughout the pandemic. This would imply that, even when included in international travel restrictions, transborder mobility affects incidence in border areas. Similarly, the incidence curves relating to intranational regional and community borders are analyzed. It is hypothesized that weak mobility leads to a clear distinction in incidence curves between both sides of these borders, at certain times during the pandemic (autumn 2020, autumn 2021, and winter–spring 2022), when the corresponding national curves differed considerably. The study is limited to the period October 2020 to December 2021. During the first wave of the pandemic, the test capacities were limited, and the reported incidences were not representative of the true incidences. This period is therefore excluded from the analysis. In addition, the first part of 2022, with many European countries reporting the highest incidences so far, is not included in this paper. Studying the different health measures and their relationship to the incidence curves in Belgium and its neighboring countries during this period is a topic for further research.

In Section 2, the study area and data are described. In Section 3, the results are discussed for The Netherlands, France, Germany, and Luxembourg, respectively. The results for the intranational language border are also discussed. We present the conclusions in Section 4.

## 2. Materials and Methods

The course of the COVID-19 pandemic from October 2020 to the end of 2021 was studied in the border areas of Belgium and its neighboring countries (The Netherlands in the north, Germany and Luxembourg in the east, and France in the south). Belgium is a small (30,688 km^2^), densely populated (375 inhabitants/km^2^) country with a long (1445 km) international border, with 56% of the population living less than 25 km from an international border [12]. Following the Schengen Agreement in 1995, Belgian border controls were abolished, and cross-border relations intensified. As a result, in the border regions, international travel and daily transborder travel are intertwined.

Belgium is a federal state, composed of communities and regions. The three geographical regions (Flanders, Brussels, Wallonia) have their own legislation, Regional Parliament, and Regional Government. Furthermore, the country consists of three language communities (the Dutch-speaking Flemish community, the French community, and the German community). Brussels is bilingual (French and Dutch), while Wallonia is French-speaking apart from the small (less than 1% of the national population) German community. The communities have powers relating to culture (theater, libraries, audiovisual media, etc.), education, the use of languages, and matters relating to the individual which concern on the one hand health policy (curative and preventive medicine) and on the other hand assistance to individuals (protection of youth, social welfare, aid to families, immigrant assistance services, etc.). The Flanders and Walloon Regions are each subdivided into five provinces. To avoid fragmentation in the fight against the virus, a Concertation Committee was set up, where the governments decided on nationwide measures [13]. The differences between the regions in Belgium are thus not linked to widely different measures, as opposed to the different measures applied in neighboring countries [14]. Indeed, there were only three differences, applying for a limited amount of time: (a) the Province of Antwerp established strict measures in late July and early August 2020 (including a curfew) to counteract the rapidly rising incidence in this province; (b) the nationwide curfew established in the autumn of 2020 started at midnight in Flanders but at 10 pm in Brussels and Wallonia; and (c) the establishment of the Covid Safe Ticket occurred at different points in time in the autumn of 2021.

On 20 March 2020, in an attempt to mitigate the viral spread, the Belgian border was closed, and border controls were temporarily reintroduced. It was prohibited for anyone to cross the border who did not have a reason mentioned explicitly on an exemption list (such as transborder employment). This measure had far-reaching consequences for the inhabitants of border municipalities. From one day to the next, they were confronted with closed border crossings and identity checks. Such measures are particularly invasive when the international border runs through a municipality’s built-up area, such as in Putte. A notorious case is Baarle-Hertog, a Belgian exclave intertwined with the Dutch municipality of Baarle-Nassau, with the border crisscrossing through houses and shops. During times when the health measures were different on both sides of the border, this led to confusing situations, with neighbors on either side of the border having to follow different rules.

During the first wave of COVID-19 in 2020, the NPIs in Belgium and its neighboring countries were similar, with all countries closing schools, restaurants, and bars, enforcing working from home, and limiting social contacts. There were, however, some differences, such as non-essential shops being closed in Belgium during the first lockdown, while they could remain open in The Netherlands provided that social distancing among customers could be guaranteed. Nevertheless, as the borders were closed during this period, this could not lead to cross-border traffic.

In the autumn of 2020, the number of infections rose again, and new lockdowns were implemented all over Europe. The national measures were not coordinated between countries, leading to different timings and stringencies in the second lockdown [15]. On the Dutch–Belgian border, for example, there were weeks where non-essential shops, bars, and restaurants were closed in Belgium but open in The Netherlands and vice versa, leading to recreational border traffic. Arguably, this had an impact on the epidemic curves in the border regions.

The COVID-19 incidences in Belgium were monitored on a daily basis by Sciensano, the national public health institute for human and animal health. The daily numbers of infections were reported for each Belgian municipality [16]. Based on these data, the 14-day incidence rates per 100,000 inhabitants are calculated for each municipality, province, and region, in addition to the national figures.

**Figure 1:**
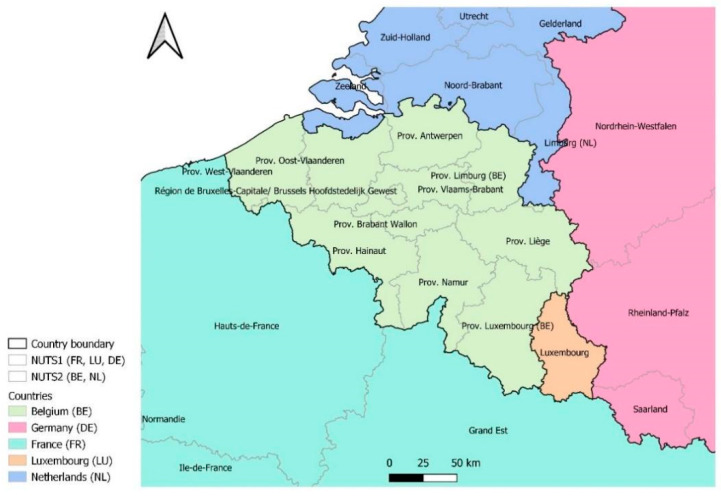
The subnational (NUTS) areas considered for analysis in Belgium and its neighboring countries.

The 14-day incidence rates per 100,000 inhabitants for the neighboring countries are based on data reported by the European Centre for Disease Prevention and Control [17]. This dataset contains the incidence rates on a subnational NUTS1 or NUTS2, level. For The Netherlands, NUTS consists of the provinces, with Zeeland, North Holland, and Limburg sharing a border with Belgium. For France, the regional level consists of the regions, with Grand Est and Hauts-de-France sharing a border with Belgium. To study the area in Belgium close to the French city of Lille, data published by Santé Publique France were used [18]. For Germany, NUTS consists of the states, with Nordrhein-Westfalen and Rheinland-Pfalz sharing a border with Belgium. Luxemburg is a NUTS area on its own. The different regional levels considered are shown in Figure 1.

Data on work-related border traffic, travel rates, the proportion of people with a different nationality, the stringency index of the NPIs, and the degree of urbanization on the level of the municipality are used as explanatory variables for incidence close to the borders.

The data on work-related border traffic, summarized in Table 1 and Table 2, are calculated based on the outgoing commuters per municipality reported by Steunpunt Werk [19] and the fraction of the population living less than 20 km from the border. The largest group of people working in a neighboring country but living in Belgium are those working in The Netherlands and Luxembourg, with the majority of them living in the Belgian provinces of Limburg and Luxembourg, respectively. On the other hand, the largest group of people living in a neighboring country but working in Belgium live in France, with the majority of them working in the province of Hainaut.

**Figure 2:**
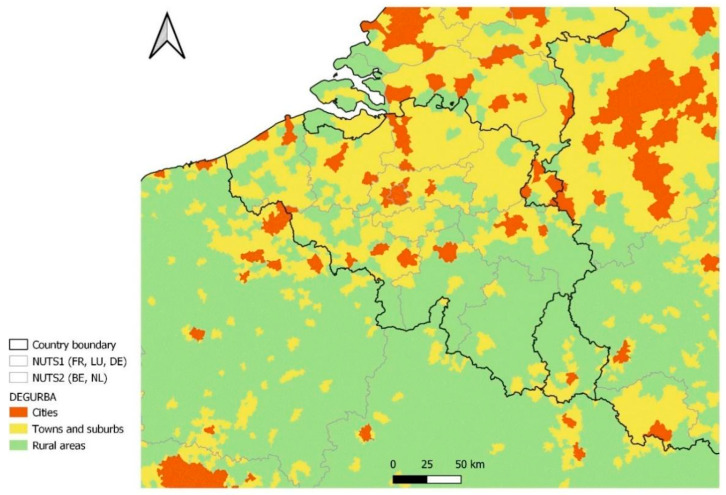
Degree of urbanization in Belgium and the neighboring countries.

The degree of urbanization (DEGURBA) and the Functional Urban Areas (FUA) classification published by EUROSTAT [20] according to a methodological manual [21] are used to identify contiguous cross-border functional areas. Cities are defined as densely populated areas when at least 50% of the population lives in urban centers. Towns and suburbs are defined as intermediate-density areas when less than 50% of the population lives in rural grid cells and less than 50% of the population lives in urban centers. Finally, rural areas are defined as thinly populated areas when more than 50% of the population lives in rural grid cells [21]. The DEGURBA is shown for Belgium and the border areas in Figure 2. The border with The Netherlands mostly consists of areas of high or intermediate density, while the border area with France, Germany, and Luxembourg mostly consists of rural areas. An exception is the area around the large French city of Lille, lying next to the border with West Flanders and Hainaut.

**Figure 3:**
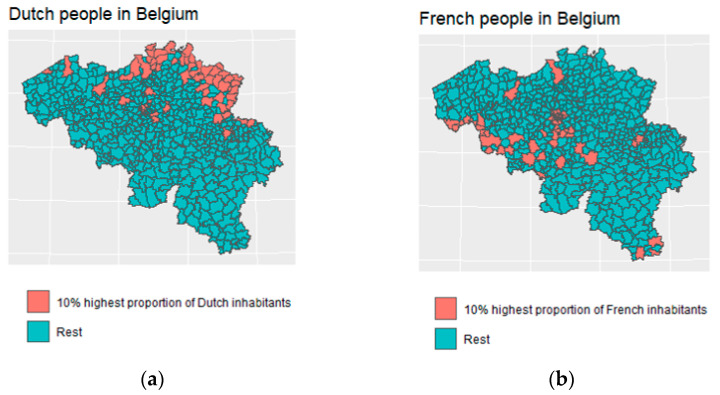
Municipalities with the 10% highest proportion of inhabitants with (**a**) Dutch nationality and (**b**) French nationality.

Data on the nationalities of people living in Belgium per municipality are reported by Statbel [22]. People with French (170,000) and Dutch (159,000) nationalities represent the largest group of foreign nationals in Belgium, followed by Italians (156,000) and Portuguese (105,000). The numbers for Belgium’s neighboring countries are summarized in Table 3. In Figure 3, the municipalities with the 10% largest proportion of people with Dutch and French nationality are shown. The municipalities with the highest proportion of Dutch nationals are situated alongside the Dutch border in Antwerp and Limburg, while the municipalities with the highest proportion of French nationals are in Brussels and close to Lille.

**Figure 4:**
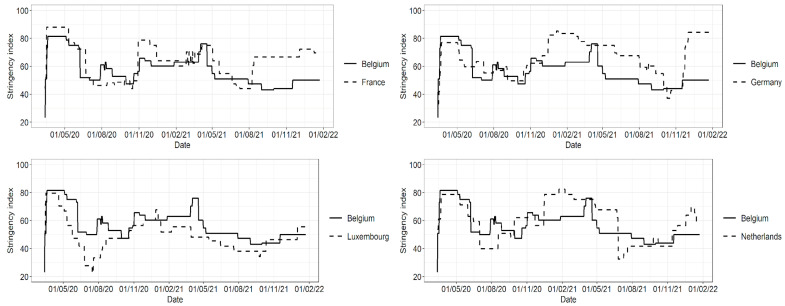
Stringency of non-pharmaceutical interventions (NPIs) for Belgium and its neighboring countries from the start of the COVID-19 pandemic in March 2020 until January 2022. Higher values indicate stricter NPIs in that period.

**Figure 5:**
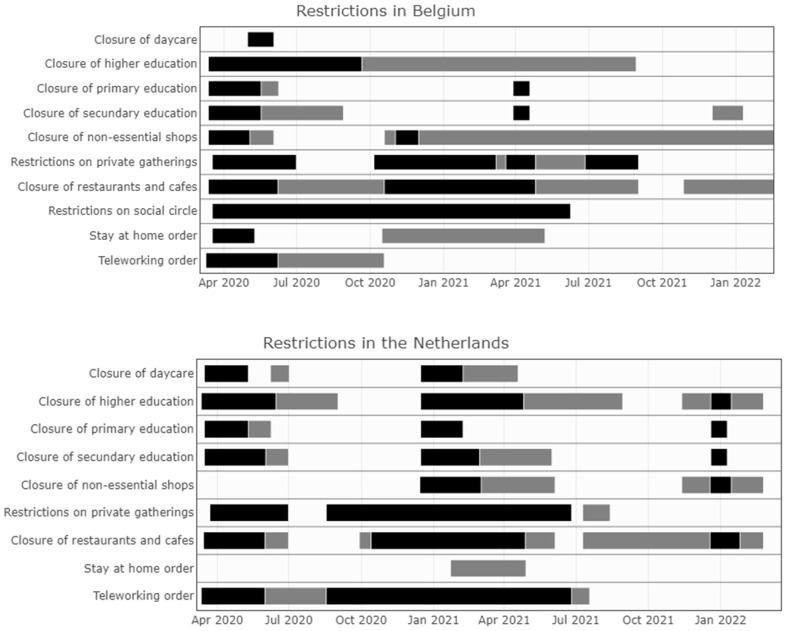

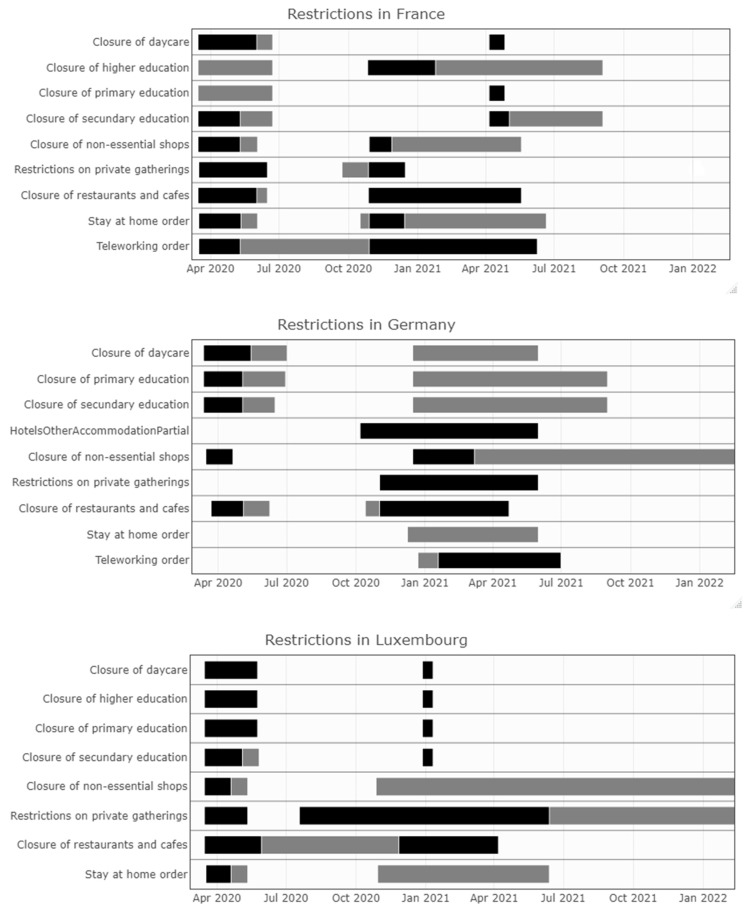
Non-pharmaceutical interventions (NPIs) implemented over time in Belgium, France, The Netherlands, Germany, and Luxembourg, based on the Response Measures Database (RMD) of the European Centre for Disease Prevention and Control (ECDC) and the Joint Research Centre (JRC) of the European Commission, from the start of the COVID-19 pandemic in March 2020 until January 2022. Black indicates full closures/restrictions, while grey indicates partial closures/restrictions.

The stringency index is a composite measure with a value from 0 to 100 (with 100 being the strictest), developed by the Oxford Coronavirus Government Response Tracker. The data were downloaded from Our World in Data [23]. For Belgium and its neighboring countries, the stringency index is shown in Figure 4. The stringency index in Belgium is systematically lower than in Germany, but higher than in Luxembourg. Alternating periods of higher and lower stringency are seen when a comparison is made with France and The Netherlands. In Figure 5, a visualization is provided for a subset of NPIs implemented over time in Belgium and its neighboring countries, based on the Response Measures Database (RMD) of the European Centre for Disease Prevention and Control (ECDC) and the Joint Research Centre (JRC) of the European Commission [24].

**Figure 6:**
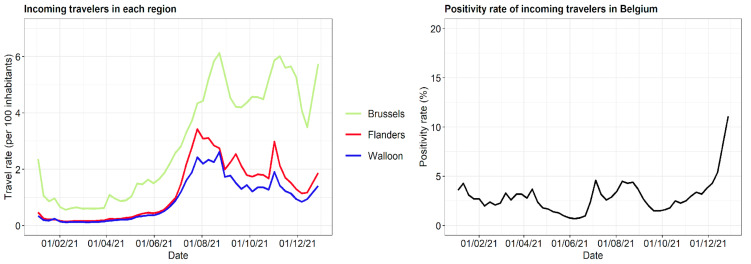
Weekly incoming travelers and COVID-19 positivity rates of incoming travelers in Belgium.

The data on the travel rate (Sciensano), i.e., the number of weekly incoming travelers per 100 inhabitants, and the positivity rate of incoming travelers doing a PCR test from January 2021 onwards are shown in Figure 6. Note that there are no reference data from before the pandemic. In Belgium, a ban on non-essential travel was in place from 27 January 2021 until 19 April 2021, leading to relatively low travel rates during this period. Another trend is visible during the second half of 2021. The periods of school holidays in summer, autumn, and around Christmas are associated with higher travel rates. Furthermore, the travel rate in Brussels was higher than in Flanders or Wallonia, as expected given the metropolitan character of the city region and its European and international mission.

The primary vaccination rate (at the end of 2021) was relatively close to 75% in the countries and regions studied. This was true for Grand Est (75%) and Hauts-de-France (75%) in France, Germany as a whole (78%), Belgium as a whole (79%), and Luxembourg (73%). Exceptions were The Netherlands as a whole (69%) and the Brussels Region (62%) at the low end and the Flemish Region (84%) at the high end.

We performed a time-trend analysis of the 14-day COVID-19 confirmed case incidence at the level of Belgian provinces, well-defined border areas within Belgium, and adjacent regions in the neighboring countries. With the aid of line charts and representative maps, we assessed the relationship of these incidences with work-related border traffic, travel rates, the proportion of people with a different nationality, the stringency index of the non-pharmaceutical interventions, and the degree of urbanization at the level of the municipality. Data processing and analysis were performed in R 4.1.3, available from the Comprehensive R Archive Network (CRAN) at https://CRAN.R-project.org/ (accessed on 1 April 2022).

## 3. Results

### 3.1. Border with The Netherlands

#### 3.1.1. Description of the Border Area

The border between Belgium and The Netherlands runs along four of the Flemish provinces: approximately, West Flanders and East Flanders share a border with the Dutch province of Zeeland, Antwerp shares a border with the Dutch province of Noord-Brabant, and Limburg shares a border with the Dutch province of Limburg. To avoid confusion, we will refer to these as BE-Limburg and NL-Limburg, respectively.

The border areas between Belgium and The Netherlands have strong economic and social bonds. Inhabitants on both sides of the border speak the same language, facilitating visits for social purposes and leisure activities, as well as cross-border commuting to work and school. Furthermore, around 159,300 Dutch people live in Belgium, of whom most live in Antwerp (64,800) and BE-Limburg (46,800). A large group of Dutch people live in the border area with Belgium, as is apparent from Figure 2, resulting in family-related border traffic. Transborder traffic restrictions may or may not have included exemptions for family-related visits (e.g., to take care of an elderly relative) in addition to restrictions for work or school purposes. In the east (Antwerp and BE-Limburg), both sides of the border have similar degrees of urbanization, while in the west (East- and West-Flanders), the Dutch border regions are more rural.

Furthermore, there are around 27,000 people living in Belgium but working in The Netherlands, of whom the majority live in BE-Limburg (16,300) and Antwerp (7900), and 12,000 people living in The Netherlands but working in Belgium, of whom the majority work in Antwerp (5000) and East Flanders (3400) (See Table 1 and Table 2).

In The Netherlands, the second lockdown started on 15 December 2020 and lasted until 19 January 2021. During these weeks, social contacts were limited, teleworking was mandatory, and schools, bars, restaurants, and non-essential shops were closed. In Belgium, this second lockdown started earlier, with non-essential shops being closed from 2 November 2020 until 1 December 2020. The different timing of the closure of non-essential shops led to considerable border traffic during the weekends, with Belgians crossing the border for leisure shopping in The Netherlands in November 2020 and vice versa in December 2020 and January 2021. As expected, the mostly Flemish cities close to the border with The Netherlands, such as Antwerp and Genk, reported an influx of Dutch visitors. One year later, in December 2021, a similar situation occurred, when relatively stringent measures were imposed in The Netherlands, closing (among other things) non-essential shops, bars, restaurants, and hotels, while this was not the case in Belgium.

#### 3.1.2. Incidence Curves

**Figure 7:**
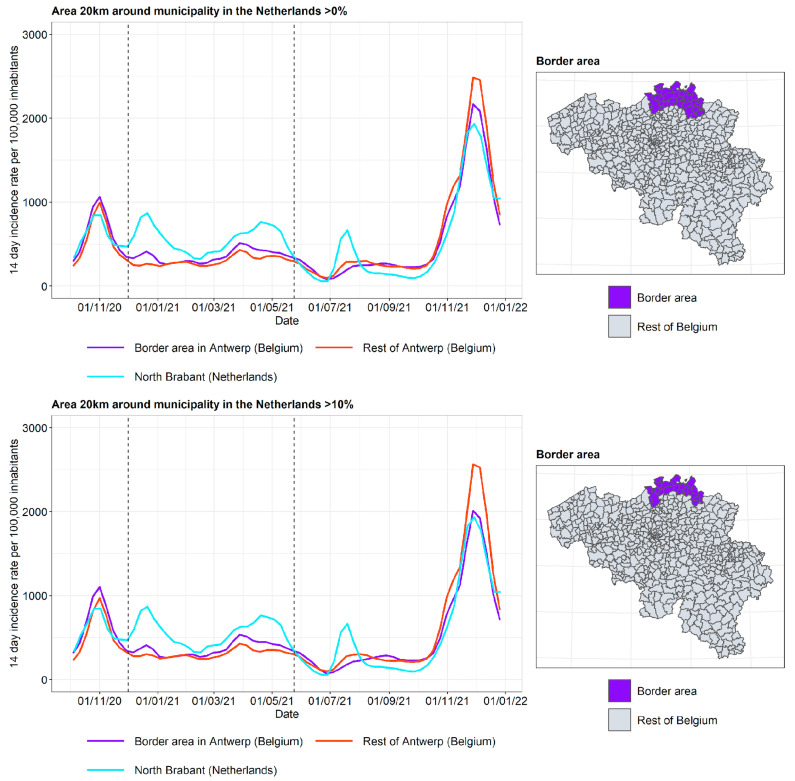
COVID-19 incidence curves for the border area of Antwerp and North Brabant. Selected period for the summary measures in Figure 10 is denoted by vertical dashed lines.

**Figure 8:**
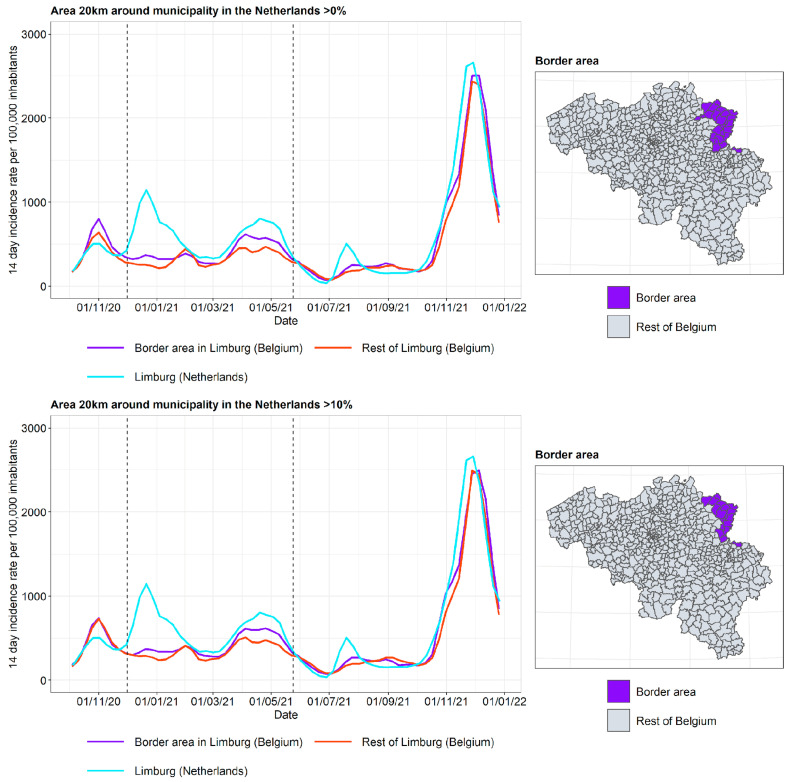
COVID-19 incidence curves for the border area of Limburg (Belgium) and Limburg (The Netherlands). Selected period for the summary measures in Figure 10 is denoted by vertical dashed lines.

**Figure 9:**
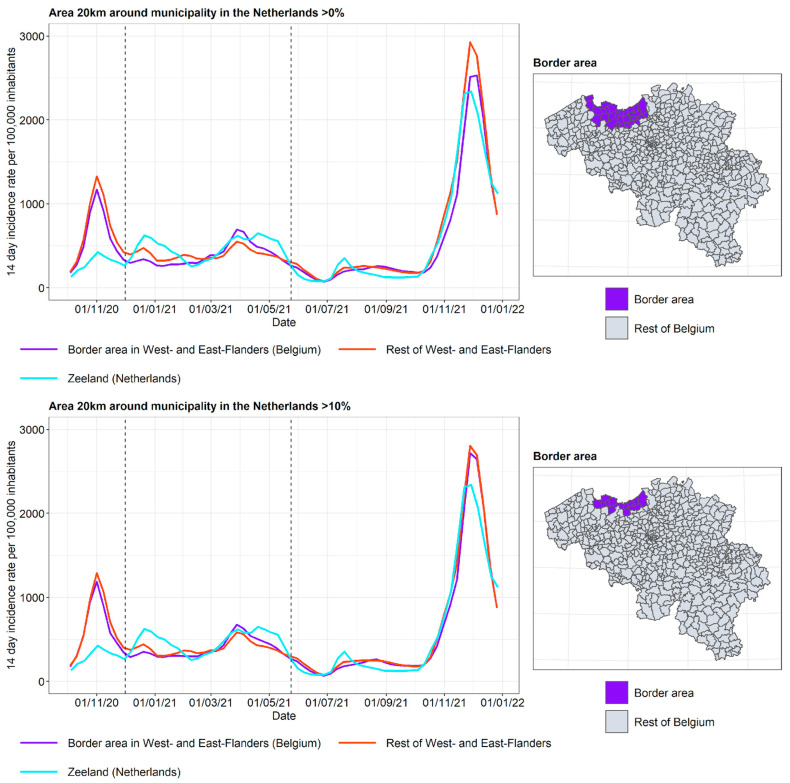
COVID-19 incidence curves for the border area of West and East Flanders and Zeeland. Selected period for the summary measures in Figure 10 is denoted by vertical dashed lines.

As each province has its own border characteristics, the incidence curves are discussed separately (see Figure 7, Figure 8 and Figure 9).

The incidence curves are plotted for the border area of the Belgian province, the rest of the Belgian province, and the neighboring Dutch province. Border area membership is determined based on the proportion of the area in a radius of 20 km around the municipality that is located in The Netherlands. The incidence curves are plotted for the border area where the proportion of the area in a radius of 20 km around the municipality that is located in The Netherlands is 0% (wide border area) and 10% (narrow border area), respectively. In this way, the gradient of the impact of the incidence curve on the other side of the border can be examined.

In Antwerp and BE-Limburg, it can be observed that the incidence curve in the border area is affected by the incidence curve in the neighboring province in The Netherlands, particularly for the period of autumn 2020 until spring 2021. Furthermore, the incidence curve in the border area is closer to the incidence curve in the neighboring province in The Netherlands when the narrow border area is considered. In this period, the second lockdown took place in both Belgium and The Netherlands, but with different stringencies and timing, as discussed previously. Non-essential travel was prohibited, but crossing the border for work and school, mostly by people living in the border area, was allowed. On 18 April 2021, the Belgian ban on non-essential travel that had existed since late January 2021 was lifted. It is hypothesized that after this relaxation, border traffic increased mostly for people not living in the border area, and therefore the difference between the incidence in the border area and the rest of the Flemish province was reduced.

The provinces of West and East Flanders share a border with the Dutch province of Zeeland. In contrast to what was observed for Antwerp and BE-Limburg, the incidence curves of the border area lie close to the incidence curves of the rest of West and East Flanders. There may be various reasons for this. Firstly, Zeeland consists of a number of islands and peninsulas, separated by two large seaports. This results in Zeeland being geographically more disconnected from Belgium than is the case for North Brabant and Limburg. Secondly, Zeeland is the least populous province of The Netherlands and has a lower degree of urbanization (see Figure 2). As it is expected that more border traffic takes place between urbanized areas, this contributes to the lower influence of the incidence curves of Zeeland on the incidence curve of the border area in West and East Flanders.

**Figure 10:**
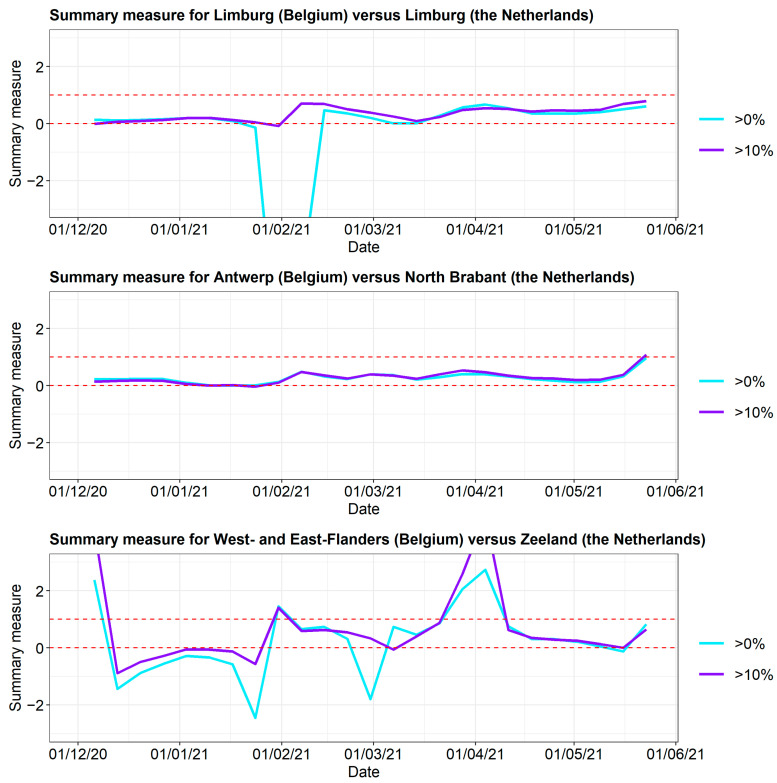
Summary measure of the COVID-19 incidences for 3 provinces along the border with The Netherlands. The red dashed lines represent *T = 1* (the incidence in the border area equals the incidence in the Dutch province) and *T = 0* (the incidence curve in the border area equals the incidence curve in the rest of the Flemish province). When the incidence curve in the border area lies between the incidence curves of the rest of the Flemish province and the Dutch province, T lies in the unit interval. When the behavior of the border region does not interpolate between its neighbors, T lies outside the unit interval.

To summarize the three curves into a single measure, the following summary measure was used: $T=\frac{B-F}{N-F}$, where *B* is the incidence in the border area in Belgium close to the border with The Netherlands, *F* is the incidence in the rest of the Flemish province, and *N* is the incidence in the adjacent Dutch province. When the incidence curve in the border area lies between the incidence curve of the rest of the Flemish province and the Dutch province, *T* lies in the unit interval. When the incidence in the border area equals the incidence in the Dutch province, *T* = 1. When the incidence curve in the border area equals the incidence curve in the rest of the Flemish province, *T* = 0. Finally, when the behavior of the border region does not interpolate between its neighbors, T lies outside the unit interval. The plots of the summary measure for the three border areas are displayed in Figure 10. The results are limited to the time frame from 1 December 2020 to 1 June 2021, as this is the period where the incidence curve in the border area appears to be influenced by the incidence curve on the other side of the border. It is indeed clear that for BE-Limburg and Antwerp, the summary measure lies between 0 and 1 for most of the time frame, which is not the case for West and East Flanders. As expected, the summary measure for a narrow border area (>10%) was larger than the summary measure for a wide border area (>0%) during most of this time frame.

### 3.2. Border with France

#### 3.2.1. Description of Border Area

The border between Belgium and France runs along four of the Belgian provinces: West Flanders and Hainaut share a border with the French region Hauts-De-France, and Namur and Luxemburg share a border with the French region Grand Est. West Flanders is located in Flanders, and the other three are in Wallonia.

The border area between Belgium and France consists of both densely populated and rural areas. In the north, the French city of Lille lies close to the border. It is hypothesized that the proximity of Lille causes considerable border traffic, and the incidence of the border area in Belgium close to Lille is influenced by that in Lille. In the south, the border area consists almost exclusively of rural areas. Furthermore, on the French side of the border, three regional parks (Parc Scarpe-Escaut, Parc de l’Avesnois, and Parc des Ardennes) form a natural separation between the two countries. For this area, it is hypothesized that the incidence curve in the border area in Belgium is not heavily influenced by the incidence curve at the French side of the border.

There are around 170,300 French people living in Belgium, of whom most live in Brussels (65,000) and Hainaut (47,000). Compared to The Netherlands, fewer French people live in the border area with France (see Figure 1). Furthermore, there are around 7300 people living in Belgium but working in France, of whom the majority live in Hainaut (6000). Furthermore, 46,300 people live in France and work in Belgium, the majority of whom work in Hainaut (20,900) and West Flanders (13,400) (See Table 1 and Table 2).

In France, the first lockdown was among the strictest in Europe, with inhabitants only being allowed to leave the house for essential reasons or for a walk within a radius of 1 km of their home address. Furthermore, a signed and dated declaration had to be carried by anyone in the public domain. The stringency index in France during the first lockdown was 88, while it was 85 in Germany, 81 in Belgium, 79 in Luxembourg, and 79 in The Netherlands. On 30 October 2020, a second nationwide lockdown began. Schools remained open, but non-essential businesses had to close, and a full stay-at-home mandate was in place until it was lifted on 15 December 2020, although even then a nationwide curfew remained in place. On 3 April 2021, a third period with a nationwide stay-at-home mandate began, including a 10 km perimeter that one could leave only with a valid reason. During this period, schools were closed for 3 weeks. From 3 May 2021 onwards, the lockdown restrictions were progressively lifted. Throughout the pandemic, France implemented a set of regional NPIs in addition to the national measures.

#### 3.2.2. Incidence Curves

**Figure 11:**
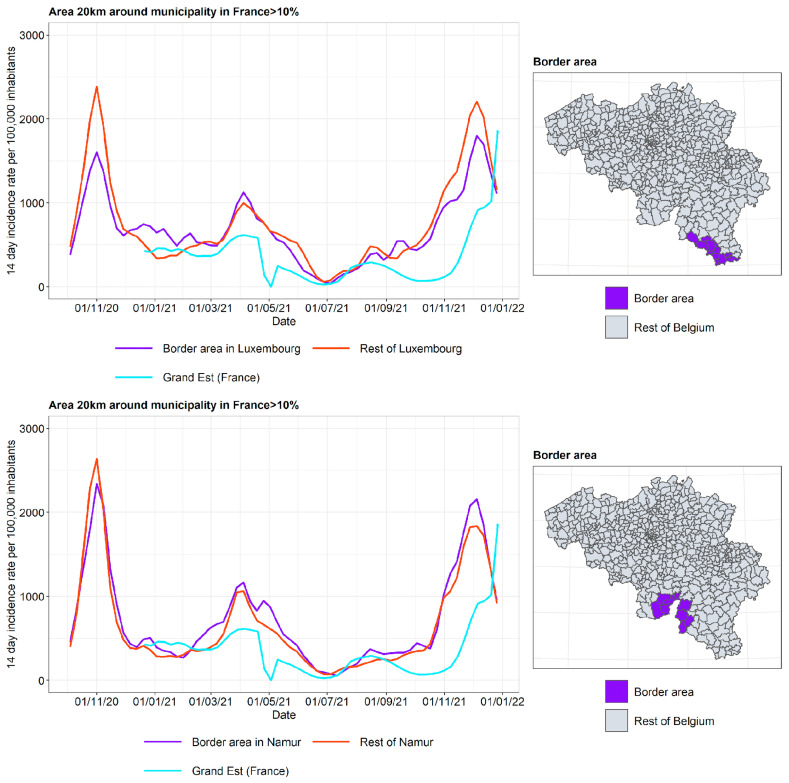
COVID-19 incidence curves for the border area with Grand Est (France).

**Figure 12:**
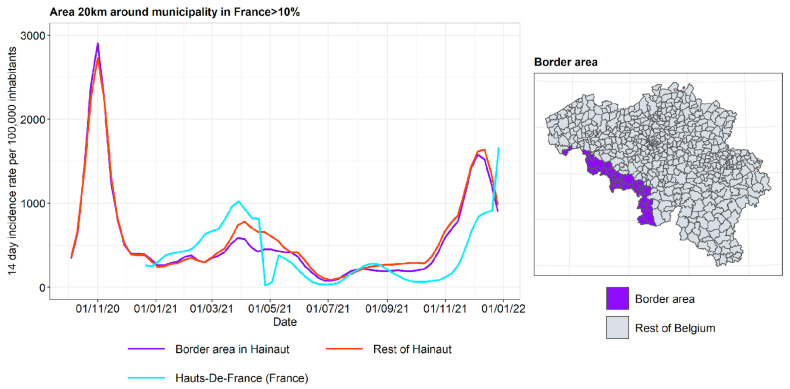

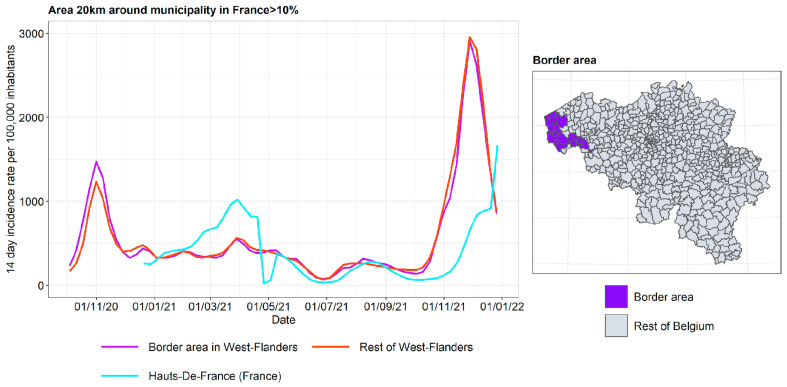
COVID-19 incidence curves for the border area with Hauts-De-France (France).

The incidence curves are plotted for the border area in the Belgian province, the rest of the Belgian province, and the French region on the other side of the border (Figure 11 and Figure 12). The incidence curves are plotted for the border area where the proportion of the area in a radius of 20 km around the municipality that is located in France is 10%. It is concluded that the incidence curve in the Belgian border area lies very close to that in the rest of the Belgian provinces, and the impact of the incidence in the French region on the other side of the border is not pronounced.

**Figure 13:**
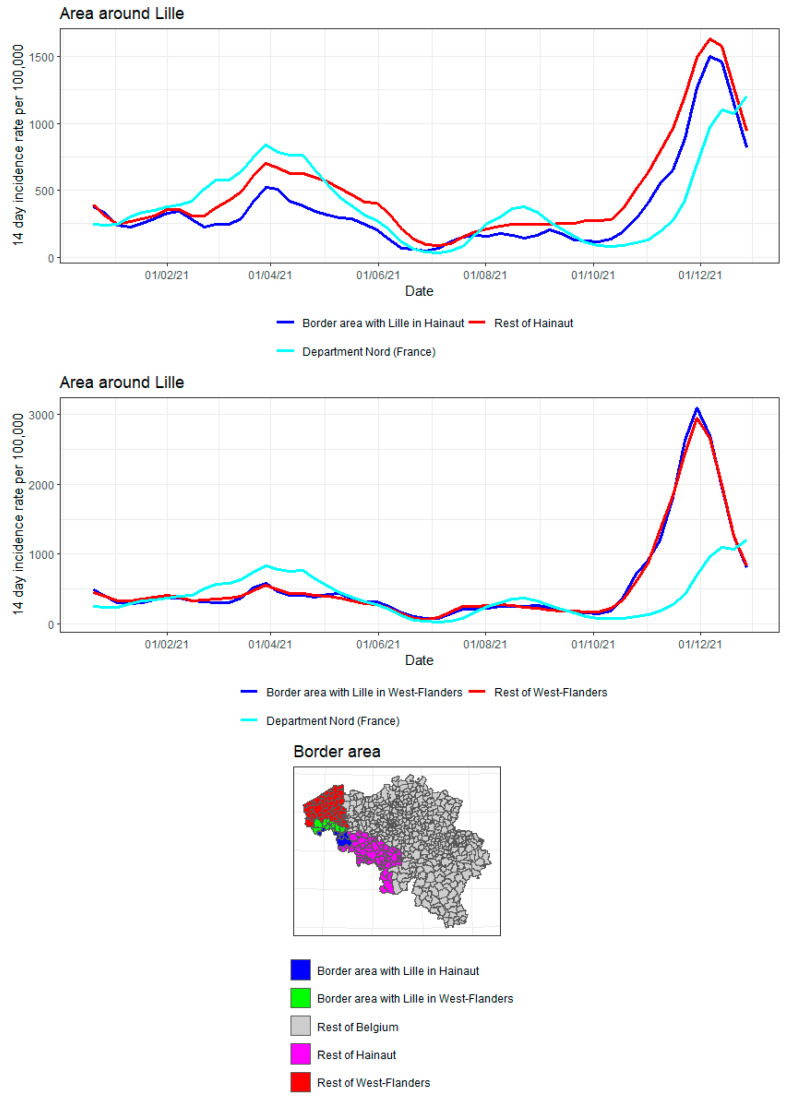
COVID-19 incidence curves for the border area around Lille (France).

As suggested before, it is expected that the city of Lille, lying very close to the Belgian border, led to considerable border traffic. Therefore, the incidence curve for the Belgian municipalities lying close to Lille are plotted separately and are compared to the incidence curve of the French department of Nord. The results are shown in Figure 13. While in West Flanders there is little or no difference between the border area and the rest of the province, the separation is somewhat more pronounced in Hainaut. Apart from the April 2021 to September 2021 peaks on the French side, the border area in Hainaut tends to produce incidences in between those of the rest of the province and the department of Nord.

### 3.3. Border with Luxembourg

The border area between Belgium and Luxembourg runs approximately along the Walloon province of Luxembourg, referred to as BE-Luxembourg. The national language in Luxembourg is Luxembourgish, but French and German are also used for administrative matters.

There are approximately 4500 Luxembourgish people living in Belgium, of whom the majority live in BE-Luxembourg (1900) and Brussels (1000). A remarkably large number of people (38,200) live in Belgium but work in Luxembourg, of whom the majority live in BE-Luxembourg (31,200) (See Table 1 and Table 2). This phenomenon is related to the fact that housing prices are much higher in the Grand Duchy of Luxembourg compared to BE-Luxembourg. On the other hand, only 500 people live in Luxembourg but work in Belgium. The large number of cross-border workers has the potential to result in a high impact of the Grand Duchy of Luxembourg incidence on that of the border region in BE-Luxembourg, including the cities of Arlon and Bastogne.

**Figure 14:**
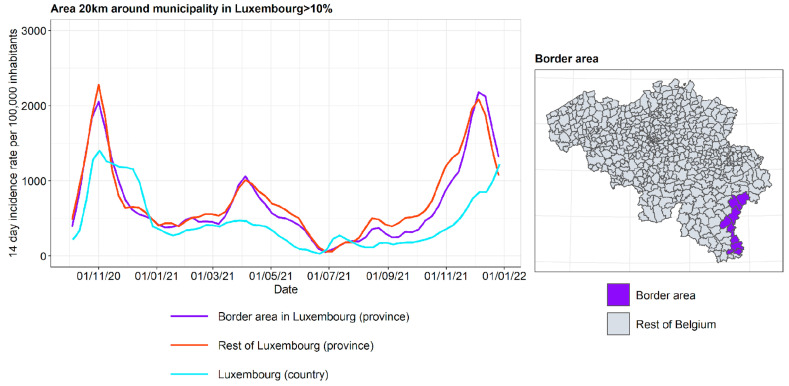
COVID-19 incidence curves for border area in BE-Luxembourg.

The incidence curves are plotted for the border area in BE-Luxembourg, the rest of BE-Luxembourg, and Luxembourg (Figure 14). The incidence curves are plotted for the border area where the proportion of the area in a radius of 20 km around the municipality that is located in Luxembourg is 10%. It is concluded that the incidence curve in the border area lies very close to the incidence curves in the rest of BE-Luxembourg, and the impact of the incidence curves in Luxembourg on the other side of the border is, contrary to expectation, relatively small, apart from a moderate effect in the autumn of 2021.

### 3.4. Border with Germany

The border between Belgium and Germany runs approximately along the Walloon province of Liège on the one hand and the German states of Nordrhein-Westfalen and Rheinland-Pfalz on the other. Along the German border lies the Belgian German-speaking community (also known as Ostbelgien), with around 78,000 inhabitants.

There are approximately 39,700 Germans living in Belgium, which is a small number compared to the 170,300 French and 159,300 Dutch citizens. The majority of these people live in Liège (14,400) and Brussels (10,700). Furthermore, there are around 3900 people living in Belgium but working in Germany, and 1350 people living in Germany but working in Belgium. The vast majority of these people work or live in Liège (See Table 1 and Table 2).

Because Ostbelgien is very small and the border area between Germany and Belgium is mainly rural (apart from the city of Aachen), it is hypothesized that the contacts between both sides are limited and that the influence of the German incidence on the incidence in the border area is limited.

**Figure 15:**
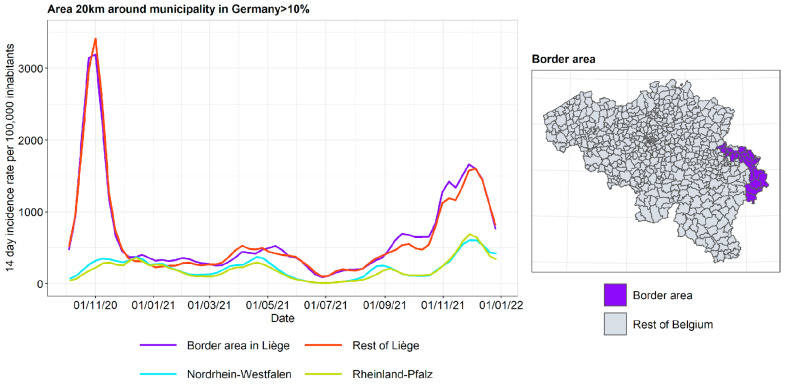
COVID-19 incidence for the border area with Germany.

The incidence curves are plotted for the border area in Liège, the rest of the province of Liège, and both German states (Figure 15). The incidence curves are plotted for the border area where the proportion of the area in a radius of 20 km around the municipality that is located in Germany is 10%. We conclude that the four incidence curves split into two pairs, often relatively separate from each other: the two German states on the one hand, and the border and non-border parts of Liège on the other. Of note is the very high peak in the province of Liège in autumn 2020, which is not mirrored in Germany. During some periods, the incidence in Ostbelgien is even further away from the German incidence than the non-border part of Liège, pointing to the fact that Ostbelgien exhibits its own dynamics.

### 3.5. Intranational Borders

The border between Flanders and Wallonia is also known as the linguistic border. The Brussels Region is an enclave in Flanders, close to the linguistic border. Although there was never a closure of the border between Flanders and Wallonia, the difference in language results in reduced contacts between the inhabitants of these regions, including social contacts as well as professional and school-related contacts. It is therefore unsurprising that the incidence on the Flemish side of the language border behaves differently from that in Wallonia, especially during peak periods.

**Figure 16:**
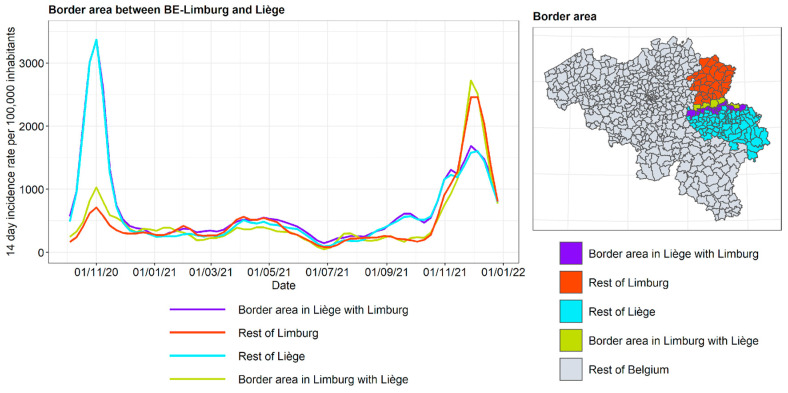
COVID-19 incidence curves for intranational language border between Liège and Limburg.

**Figure 17:**
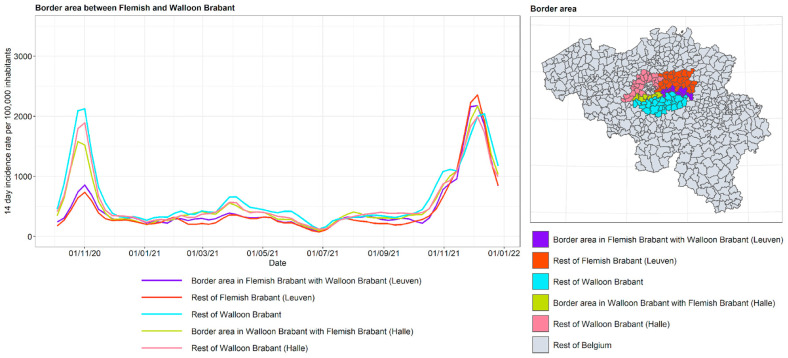
COVID-19 incidence curves for intranational language border between Flemish Brabant and Walloon Brabant.

**Figure 18:**
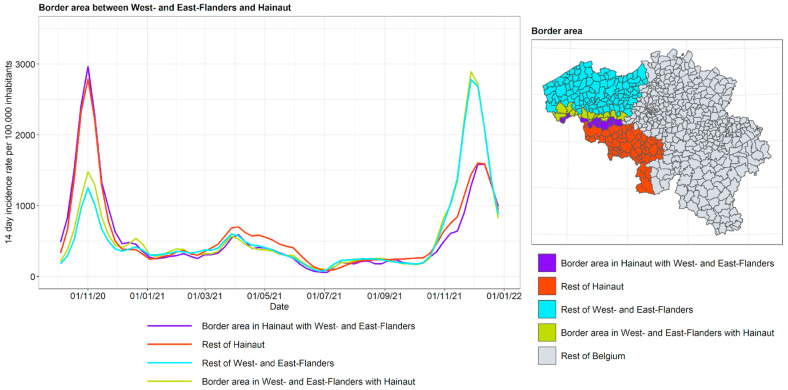
COVID-19 incidence curves for intranational language border between Hainaut and West and East Flanders.

**Figure 19:**
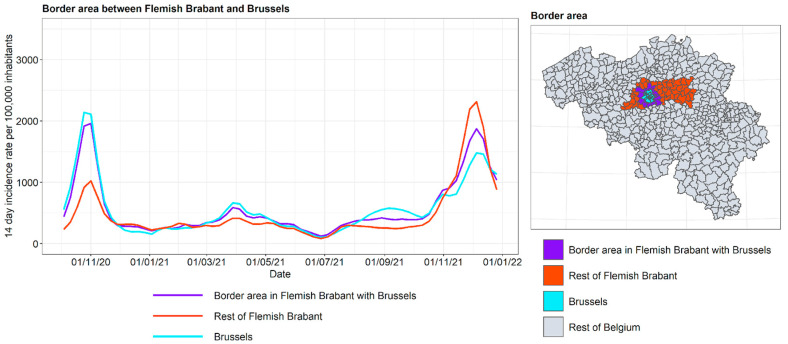
COVID-19 incidence curves for the border area in Flemish Brabant with Brussels.

The incidence curves are plotted for the border area between Flanders and Wallonia for the various neighboring provinces: in Flanders: Limburg, Flemish Brabant, East Flanders, and West Flanders; in Wallonia: Liège, Walloon Brabant, and Hainaut. For Flemish Brabant, a distinction is made between its two so-called arrondissements of Halle-Vilvoorde and Leuven. The arrondissements form an administrative division subordinate to the province. Halle-Vilvoorde surrounds the capital of Brussels and is strongly interrelated with the capital region, given that a large fraction of its population commute to Brussels and depend on the city for goods and services. It is hypothesized that in this arrondissement, the contacts with Brussels, and through Brussels with Wallonia, are higher. This motivated a separate treatment from Leuven, where the population has a stronger relationship with the rest of Flanders. The results are shown in Figure 16, Figure 17, Figure 18 and Figure 19.

For Limburg and Liège, the conclusion is very clear. The border regions in each of these two provinces follow closely the behavior of the rest of their province. At the same time, the provinces as a whole tend to follow their own incidence dynamics, which is strikingly clear in the autumn of 2020 (the large peak in Liège is about four times higher than that in Limburg) and in the autumn of 2021 (a large peak in Limburg, considerably smaller in Liège).

For the border area between West and East Flanders on the one hand and Hainaut on the other, the conclusions are somewhat similar, with the exception of autumn 2020, at which time the peak is much higher in Hainaut than in West and East Flanders and the border region in the latter provinces exhibits a somewhat higher peak. This suggests that there is slightly more contact in this part of the country than between Limburg and Liège, although the extent to which this occurs should not be overinterpreted.

For the border area between Flemish Brabant and Walloon Brabant, the incidence curves confirm that the Flemish arrondissement of Halle-Vilvoorde follows the same dynamics as the Brussels Region. Halle-Vilvoorde is very different from the rest of Flemish Brabant. In the eastern part of this region, the border region between Flemish Brabant and Walloon follows its own province. In other words, the eastern border between the two Brabant provinces has an effect similar to that between Limburg and Liège.

## 4. Conclusions

In this section, locations are prefixed with their country code to avoid confusion, except when the intra-Belgium language border is discussed.

In this study we compared the incidence curves during the COVID-19 pandemic from October 2020 to December 2021 in the border areas of Belgium with the incidence curves in the neighboring countries of The Netherlands, France, Luxembourg, and Germany. In addition, the impact on incidence of the intra-Belgium language border between Flanders and Wallonia was examined.

The clearest evidence that a border region was influenced to some extent by the neighboring region was seen between BE-Limburg and NL-Limburg, which form one contiguous cross-border functional area, and also between BE-Antwerp and NL-North Brabant. The effect was less pronounced between both BE-West Flanders and BE-East Flanders, and NL-Zeeland. NL-Zeeland is more rural, sparsely populated, and relatively isolated from Belgium and the rest of The Netherlands, due to the main rivers that act as natural barriers.

The influence of France on the Belgian border regions was visible near the metropolis of FR-Lille and was greater on the border region in BE-Hainaut than in BE-West Flanders. The rest of the border region between France and Belgium comprises rural areas and natural parks, and consequently there was less influence.

The Grand Duchy of Luxembourg had a visible impact on the incidence in the border region of BE-Luxembourg, especially in autumn 2021. In this case, cross-border travel is not related to a high degree of urbanization. A large number of people live in this rural and forested Belgian province and work in Luxembourg.

Germany did not appear to influence the Belgian border region incidence, in spite of the presence of the germanophone Ostbelgien. BE-Liège, including its border region, had a huge peak in autumn 2020, with no peak at all in the German states of Nordrhein-Westfalen and Rheinland-Pfalz. Moreover, in late 2021, Ostbelgien was even further away from the German states in terms of incidence than the rest of BE-Liège.

The intra-Belgian language border, running between Flanders and Wallonia, with Brussels nearby in the center of the country, showed a variety of border effects. The urbanized area surrounding Brussels within Flanders (Halle-Vilvoorde) had an incidence curve very similar to that of Brussels. The language border east of Brussels was clear in the COVID-19 incidence curves: the border regions followed the incidence of their own provinces on the one hand, with the adjacent provinces north and south of the language border often having very different incidences, especially in the autumn of 2020 (much higher incidence in Wallonia) and the autumn of 2021 (much higher incidence in Flanders). To the west of Brussels, the language border was slightly less impenetrable, with the border region of East and West Flanders influenced by Hainaut in autumn 2020. Early in 2021, the border region in Hainaut followed East and West Flanders, with the rest of Hainaut exhibiting higher incidence.

During certain periods, the NPIs in place in Belgium and its neighboring countries were different (e.g., the closure of non-essential shops, restaurants, and bars in The Netherlands at the end of 2021). Especially in urbanized transnational regions, regions with many citizens from the neighboring countries in residence, or regions with considerable work- and school-related transborder commuting, coordination of NPIs is important to avoid intensifying contacts even further at times when it is epidemiologically necessary to reduce contacts. While this advice seems self-evident, the packages of NPIs in the various countries underscore the fact that decisions have sometimes been taken otherwise.

At the same time, such coordination is less urgent in regions with limited transborder contact, such as in rural zones (unless they are residential areas with transborder commuting such as the forested province of Luxembourg), regions with natural barriers, or regions with cultural separation. The latter is the case, for example, over the better part of the intra-Belgium language border.

Arguably, such recommendations will be useful for policymakers at a national and local (province, department, state, municipality, etc.) level, as well as at the European level, where a framework is needed to counteract a pandemic and where guidelines for coordination between (neighboring) countries are needed. This goes hand in hand with the need for high-quality data, both at the epidemiological level (including but not limited to data on confirmed cases) and in terms of covariables such as high-quality mobility data (e.g., based on data from telecommunications operators).

A limitation is that the effect of variations in NPIs was not established using a randomized experiment. However, the fact that there were periods with very similar stringencies and others with quite different stringencies acts as a natural experiment. Further, for the intra-Belgium language border, the NPIs to its north and south were virtually always the same, except for a period in late 2020 and early 2021 when the Flemish curfew started at midnight and that in the rest of the country at 10 pm. Furthermore, the so-called Covid Safe Ticket (to assess the vaccination, testing, or recovery status of the bearer) was introduced in Flanders a few weeks later than in the rest of the country. It is important to note that intra-Belgium mobility restrictions were never imposed during the period under investigation. At the transborder level, the border was closed in March and April 2020 (outside the study period), and non-essential travel was banned from 27 January to 19 April 2021 (but still with school- and work-related mobility, as well as mobility for well-defined essential purposes allowed in the border regions) but was unrestricted otherwise. Therefore, the effects observed around the language border and Brussels are largely attributable to “natural” contact between the regions rather than to NPI-induced additional mobility.

## Figures and Tables

**Table 1:** Number of people living in Belgium but working in a neighboring country.

Outgoing Work-Related Border Traffic					
Province	Germany	France	Luxembourg	The Netherlands	Total
Antwerp	89	31	199	7916	8235
Brussels	15	69	135	75	294
Hainaut	16	5927	267	16	6226
Limburg	234	6	139	16,343	16,722
Liège	3441	63	4950	874	9328
Luxembourg	9	212	31,229	15	31,465
Namur	5	199	834	10	1048
East-Flanders	43	88	78	1338	1547
Flemish Brabant	41	26	185	316	568
Walloon Brabant	7	41	156	7	211
West Flanders	26	690	51	297	1064
Total	3926	7352	38,223	27,207	76,708

**Table 2:** Number of people living in a neighboring country but working in Belgium.

Incoming Work-Related Border Traffic					
Province	Germany	France	Luxembourg	The Netherlands	Total
Antwerp	146	376	15	5068	5604
Brussels	151	1653	99	439	2342
Hainaut	15	20,934	18	25	20,993
Limburg	178	92	6	1956	2233
Liège	650	397	56	210	1313
Luxembourg	17	6645	237	27	6925
Namur	4	1369	18	7	1397
East-Flanders	55	661	11	3434	4161
Flemish Brabant	89	368	23	375	855
Walloon Brabant	33	438	15	15	500
West Flanders	20	13,457	11	669	14,156
Total	1356	46,389	508	12,224	60,479

**Table 3:** Number of people living in Belgium with a nationality from a neighboring country.

Different Nationalities from Neighboring Countries in Belgium					
Province	Germany	France	Luxembourg	The Netherlands	Total
Antwerp	3354	3494	114	64,896	71,858
Brussels	10,703	65,656	1006	8276	85,641
Hainaut	1110	47,770	106	893	49,879
Limburg	1635	512	20	46,825	48,992
Liège	14,404	12,063	530	4220	31,217
Luxembourg	280	6972	1982	926	10,160
Namur	247	6465	132	428	7272
East-Flanders	1515	2576	63	14,296	18,450
Flemish Brabant	4526	7874	229	11,948	24,577
Walloon Brabant	1025	9974	275	819	12,093
West Flanders	914	6965	65	5794	13,738
Total	39,713	170,321	4522	159,321	373,877

## Data Availability

Only publicly available datasets were analyzed in this study. Data on Belgian COVID-19 incidences: https://datastudio.google.com/embed/u/0/reporting/c14a5cfc-cab7-4812-848c-0369173148ab/page/tpRKB (accessed on 3 June 2022). Data on COVID-19 incidences in the neighboring countries of Germany, Luxembourg, and The Netherlands: https://www.ecdc.europa.eu/en/publications-data/weekly-subnational-14-day-notification-rate-covid-19 (accessed on 3 June 2022). Data on COVID-19 incidences in France: https://www.data.gouv.fr/fr/datasets/taux-dincidence-de-lepidemie-de-covid-19/ (accessed on 3 June 2022). Data on work-related border traffic in Belgium: https://www.steunpuntwerk.be/cijfers?search=&sort=postDate%20DESC (accessed on 3 June 2022). Data on the DEGURBA: https://ec.europa.eu/eurostat/web/degree-of-urbanisation/data/database (accessed on 3 June 2022). Data on the nationalities of people living in Belgium per municipality: https://statbel.fgov.be/nl/themas/bevolking/herkomst#figures (accessed on 3 June 2022). Data on stringency indexes: https://ourworldindata.org/covid-stringency-index (accessed on 3 June 2022).
